# Characteristic and phylogenetic analyses of chloroplast genome for *Mentha haplocalyx* (Lamiaceae)

**DOI:** 10.1080/23802359.2019.1680322

**Published:** 2020-05-19

**Authors:** Shui-Lian He, Yang Yang, Yang Tian

**Affiliations:** a College of Horticulture and Landscape, Yunnan Agricultural University, Kunming, China; b Yunnan Key Laboratory of Biomass Big Data, Yunnan Agricultural University, Kunming, China; c College of Science, Yunnan Agricultural University, Kunming, China

**Keywords:** *M. haplocalyx*, medicinal and edible plant, chloroplast genome, Phylogeny

## Abstract

*Mentha haplocalyx* is an important medicinal and edible plant. Now, the complete chloroplast (cp) genome of *M. haplocalyx* was assembled and annotated. The cp genome of *M. haplocalyx* was 152,048 bp and contained two short inverted repeat regions (25,608 bp) which were separated by a small single copy region (17,654 bp) and a large single copyregion (83,179 bp). The cp genome encodes 113 unique genes, including 79 protein-coding genes, 30 transfer RNA genes and 4 ribosomal RNA genes. The topology of the phylogenetic tree showed that *M. haplocalyx and M. spicata* formed a monophyletic clade and clustered together with genus *Dracocephalum*.

*Mentha haplocalyx Briq.*, a perennial herbaceous plant of the family Lamiaceae, is widely distributed in southwest of China and popularly used in food, cosmetics and medicines. As a traditional Chinese medicine, it is clinically used to treat diseases in the nerve centre, breath, procreation and digestive systems (Su et al. 2014; He et al. 2019). The origin of the taxa is not accurately resolved and taxonomical position is still unclear. In the present study, applying the Illumina technology, the whole chloroplast genome of *M. haplocalyx* was sequenced, assembled, annotated. The resultant data have been made publicly available as a resource for genetic information for *Mentha* species, and will provide a valuable plastid genomic resource for the future genetic and phylogenetic studies about *M. haplocalyx.*

The fresh leaves of *M. haplocalyx* were collected from the field of Kunming (25.10°N, 102.76°E). The voucher specimen was deposited at Herbarium of Yunnan Agricultural University (No. 2019HSL001). Total genomic DNA was isolated from fresh leaves using a DNeasy Plant Mini Kit (QIAGEN, Valencia, California, USA) according to the manufacturer’s instructions to construction chloroplast DNA libraries. The Illumina sequencing was conducted by Biomarker Technologies Inc. (Beijing, China). Resultant clean reads were assembled using GetOrganelle pipeline (https://github.com/Kinggerm/GetOrganelle). The genome was automatically annotated by using the CpGAVAS pipeline (Liu et al. 2012) and start/stop codons and intron/exon boundaries were adjusted in Geneious R11.0.2 (Biomatters Ltd., Auckland, New Zealand). All the contigs were checked against the reference genome of *Mentha spicata* (NC037247).

The complete chloroplast genome of *M. haplocalyx* was 152,048 bp in length (Genbank accession number: MN102358). It was the typical quadripartite structure and contained contained two short inverted repeat (IRa and IRb) regions (25,608 bp) which were separated by a small single copy (SSC) region (17,654 bp) and a large single copy (LSC) region (83,179 bp). The cp genome encodes 113 unique genes, including 79 protein-coding genes, 30 transfer RNA (tRNA) genes and 4 ribosomal RNA (rRNA) genes. Twenty-one gene species are partially or completely duplicated, including ten PCG (*ndhB*; *ndhF*; *rpl2*; *rpsl23*; *rps12*; rps19; *rps7*; *ycf1*; *ycf2; ycf15*), seven tRNA (*trnI-GAU*, *trnA-UGC*, *trnL-CAA*, *trnI-CAU*, *trnR-ACG*, *trnV-GAC*, *trnN-GUU*) and all four rRNA (4.5S, 5S, 16S & 23S rRNA). The overall GC content of the cp genome was 37.8%, while that of LSC, SSC and IR regions was 35.9%, 31.9% and 43.0%, respectively.

The chloroplast genome sequences of Lamiaceae were downloaded from GenBank and aligned with *M. haplocalyx* using MAFFT (Katoh and Standley 2013) in Geneious R11.0.2. To resolve its phylogenetic placement within the family Lamiaceae, the maximum likelihood (ML) phylogeny tree was reconstructed using RA × ML version 8.1.1179 (Stamatakis 2014). *Magnolia alba* (NC037005, Magnoliaceae) was selected as the outgroup. The topology of the phylogenetic tree showed that *M. haplocalyx and M. spicata* formed a monophyletic clade and clustered together with genus *Dracocephalum* (Figure 1). The complete cp genome information reported in this study will be a valuable resource for future studies of the species.

**Figure 1.**
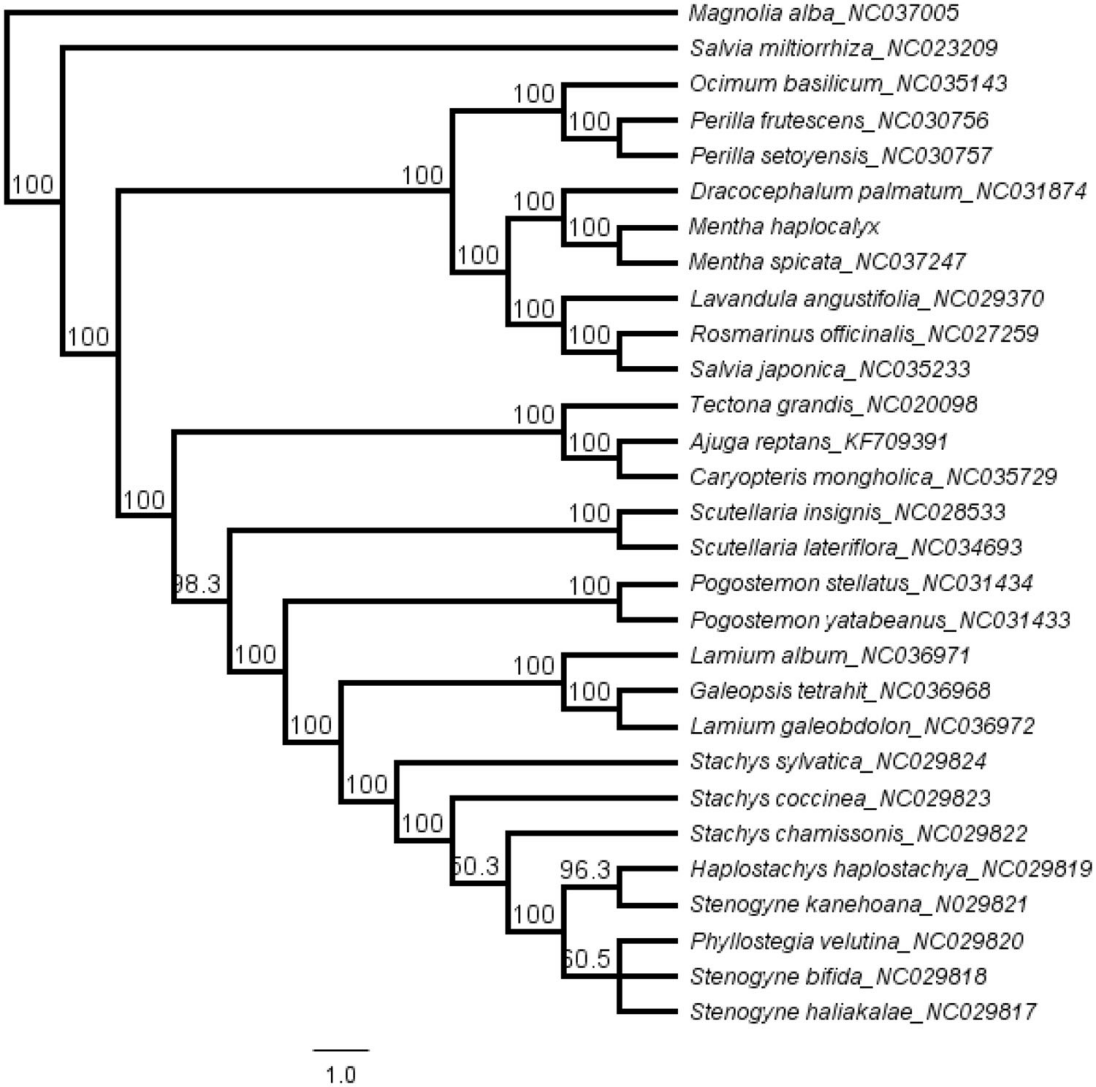
The maximum likelihood (ML) phylogenetic tree based on 31 complete chloroplast genome sequence. Numbers at the right of nodes are bootstrap support value.
